# Prophylactic Methylprednisolone in Neonatal On-Pump Cardiac Surgery

**DOI:** 10.31083/RCM49485

**Published:** 2026-06-26

**Authors:** Peng Gao, Yu Jin, He Wang, Peiyao Zhang, Wenting Wang, Jia Liu, Zhengyi Feng, Bingyang Ji, Jinping Liu, Jinxiao Hu

**Affiliations:** ^1^Department of Anesthesiology, Peking Union Medical College Hospital, Chinese Academy of Medical Sciences and Peking Union Medical College, 100007 Beijing, China; ^2^Pediatric cardiac center, Fuwai Hospital, National Center for Cardiovascular Diseases, Chinese Academy of Medical Sciences and Peking Union Medical College, 100037 Beijing, China; ^3^Department of Anesthesiology, Beijing Chao-Yang Hospital, Capital Medical University, 100020 Beijing, China; ^4^Department of Anesthesiology, Jiangsu Province Hospital, Nanjing Medical University, 210029 Nanjing, Jiangsu, China

**Keywords:** corticosteroids, neonate, cardiac surgery, cardiopulmonary bypass, clinical outcome, inflammatory response

## Abstract

**Background:**

Corticosteroids are commonly used in neonatal cardiac surgery to reduce inflammation. Previous studies have shown that administering methylprednisolone (MP) during the perioperative period can reduce the inflammatory response. However, the impact of MP on postoperative clinical outcomes in neonates remains unclear. Thus, this study aimed to assess the effects of MP on postoperative inflammation and clinical outcomes in neonates undergoing cardiac surgery involving cardiopulmonary bypass (CPB).

**Methods:**

This was a prospective, non-randomized, unblinded, controlled trial in which the clinicians determined treatment assignment. A total of 86 neonates who underwent cardiac surgery with CPB between December 2020 and August 2023 were included. After induction of anesthesia, the MP group received a single dose of 30 mg/kg MP, while the placebo group received an equal volume of saline solution. Primary endpoints were plasma interleukin (IL-6, IL-8, and IL-10) and D-dimer concentrations. Composite outcomes included death, respiratory infection, cardiac arrest, need for extracorporeal membrane oxygenation, acute kidney injury, low cardiac output syndrome, and need for prolonged mechanical ventilation.

**Results:**

Consistent with the observed anti-inflammatory effects, MP administration was associated with significantly lower levels of proinflammatory cytokines (IL-6, IL-8) and higher levels of the anti-inflammatory cytokine (IL-10). MP did not significantly reduce the likelihood of the composite outcome (*p* = 0.664), with 25 patients (58.1%) in the MP group and 23 patients (53.5%) in the placebo group experiencing such outcomes. The MP group also showed a significantly lower postoperative vasoactive–inotropic score and higher postoperative procalcitonin levels and nadir mixed venous oxygen saturation during the first 24 hours. No significant differences in postoperative D-dimer, blood glucose, or insulin administration were observed between the two groups.

**Conclusions:**

Prophylactic administration of 30 mg/kg MP in neonates undergoing cardiac surgery with CPB did not result in a statistically significant improvement in clinical outcomes compared with placebo. However, this intervention was associated with a reduction in the inflammatory response.

**The Clinical Trial Registration:**

ChiCTR 2000040230, https://www.chictr.org.cn/showproj.html?proj=64716.

## 1. Introduction

Cardiac surgery involving cardiopulmonary bypass (CPB) often triggers an acute systemic inflammatory response, often due to exposure to nonbiological surfaces within the CPB circuit, endotoxemia, and ischemia-reperfusion injury [1,2]. This systemic inflammatory response can exacerbate stress during surgery and is linked to an increased risk of negative post-surgery outcomes and organ dysfunction [3,4,5]. Over recent decades, there have been significant improvements in CPB technology, including biocompatible coatings, ultrafiltration, and miniaturized circuits. Nevertheless, pharmacological approaches, particularly glucocorticoids (GCs), remain the main method for managing the inflammatory response induced by CPB [6]. GCs are also used to support the immature adrenal function in neonates. According to the Society of Thoracic Surgeons (STS) Congenital Heart Surgery Database, over half of pediatric CPB surgeries involve the use of GCs [7]. Studies have shown that the perioperative application of methylprednisolone (MP) can decrease pro-inflammatory mediators while increasing anti-inflammatory mediators [8,9,10,11].

Since 1980, randomized controlled trials (RCTs) have been examining the role of GCs in pediatric cardiac surgery [12]. Although numerous RCTs have been conducted on perioperative prophylactic use of GCs in children’s cardiac procedures, including a major study (the STRESS trial) with 1349 infants [13,14]. The benefits of perioperative corticosteroids in pediatric cardiac surgery remain inconclusive due to variability in patient populations, agents, and dosages [15,16,17]. Fewer than ten have specifically focused on neonates, with most involving fewer than 50 participants [16,18]. There is a clear need for more extensive research on the prophylactic use of GCs in neonatal cardiac surgery. Corticosteroids, such as MP, achieve potent anti-inflammatory effects by binding to glucocorticoid receptors and altering gene expression [19]. Prophylactic use of MP, a medium-strength glucocorticoid, has been the main intervention method. Currently, the most prevalent method of administering MP involves a single dose of 30 mg/kg, given after anesthesia induction or preoperatively in the CPB circuit prime solution [17].

This study aims to evaluate whether a single prophylactic dose of 30 mg/kg MP administered after anesthesia induction can reduce inflammation and improve postoperative outcomes in neonates undergoing cardiac surgery with CPB.

## 2. Materials and Methods

### 2.1 Study Design

This research was conducted as a prospective, non-randomized controlled trial without blinding to evaluate the administration of MP in neonatal cardiac surgery, which may introduce potential indication bias. The trial included neonates aged 28 days or younger who were slated for congenital heart surgery involving cardiopulmonary bypass at Fuwai Hospital in Beijing, China. Neonates were excluded if they were premature (gestational age under 37 weeks), had severe perinatal central nervous system injuries, required mechanical ventilation, used inotropes or steroids prior to surgery, or were enrolled in other studies.

The Institutional Ethics Committee approved the study, and written informed consent was secured from the patients’ legal guardians.

### 2.2 Enrollment

All eligible participants were assigned to the MP or placebo group in a 1:1 ratio based on clinical judgment of the treating team (anesthesiologists and surgeons), aiming to balance baseline characteristics between groups. An independent pharmacist then prepared a syringe, containing either MP at a dosage of 30 mg/kg or a placebo consisting of an equivalent volume of 0.9% saline solution. Regardless of their assigned group, all patients were administered the study medication using this syringe.

### 2.3 Clinical Setting

All surgeries were conducted under general anesthesia using a combination of sufentanil, sevoflurane, midazolam, and dexmedetomidine. Cisatracurium was administered as the muscle relaxant. For anticoagulation purposes, sodium heparin was given at an initial dose of 4 mg/kg. The activated coagulation time (ACT) was monitored using the Hemochron Jr. Signature Whole Blood Microcoagulation System, aiming for a target of over 410 seconds before and during CPB-. After CPB, protamine sulfate was employed to counteract the anticoagulant effects of heparin. Additionally, tranexamic acid was infused throughout the surgery to aid in controlling bleeding.

CPBwas conducted following the established institutional perfusion protocol [20] after systemic heparinization and a standard median sternotomy. The CPB setup included a Terumo FX05 oxygenator (Terumo Corporation, Tokyo, Japan), uncoated polyvinyl chloride tubing (Tianjin Plastic Factory, Tianjin, China), and Stockert S5 roller pumps (Sorin, Munich, Germany). The circuit was primed with 40 mL of Plasma-Lyte A (Baxter Healthcare, Deerfield, IL, USA), 80 mL of packed red blood cells, 30 mL of 20% human serum albumin, and 10 mL of 5% sodium bicarbonate. The flow rate was maintained at 150–200 mL/kg/min based on the patient’s actual body temperature to achieve target levels of venous oxygen saturation (SvO_2_) ≥70%, mean arterial pressure (MAP) between 25–50 mm Hg, lactate levels below 3 mmol/L, and a base excess (BE) within the range of –3 to +3. Regional antegrade cerebral perfusion was applied during aortic arch reconstruction, maintained at 25 °C and a flow rate of 20–50 mL/kg/min, with cerebral near-infrared spectroscopy (NIRS) monitoring aimed at maintaining readings above 80% of the baseline. For myocardial protection, antegrade cardioplegia was delivered using Bretschneider’s solution (Custodiol Solution) at a rate of 50–60 mL/kg, administered cold. Deep hypothermic circulatory arrest (DHCA) was utilized at 20 °C when required. Alpha-stat management was implemented to keep hematocrit levels between 24%–27% and colloid osmotic pressure (COP) between 12–16 mm Hg during CPB. Both conventional and modified ultrafiltration were performed for all patients. After weaning from CPB through modified ultrafiltration, the COP was maintained within 18–22 mm Hg, and hematocrit was targeted at 35%–40%. All surgical procedures were conducted by a consistent team of surgeons, and patients were transferred to the pediatric intensive care unit (PICU) post-operation, under the care of the newborn critical care team.

Inotropic support involved dopamine, dobutamine, epinephrine, norepinephrine, phenylephrine, milrinone, or pituitrin. The vasoactive inotropic score (VIS) was calculated according to the method described in a previous study [21]. In the pediatric intensive care unit (PICU), the initiation of insulin infusion was determined by the attending physicians and was typically started when blood glucose levels consistently exceeded 10 mmol/L. The use of MP was not restricted and was permitted as needed, particularly in cases of suspected allergic reactions during or following surgery.

### 2.4 Data Collection

The primary objectives of this study were to measure plasma levels of inflammatory interleukins (IL-6, IL-8, and IL-10) and D-dimer, indicators of inflammation, coagulation, and fibrinolysis. Blood samples were taken post-anesthesia induction and 24 hours post-surgery for the analysis of interleukin levels. These samples were immediately processed at the institutional central laboratory, where plasma was separated by centrifugation and analyzed. The levels of IL-6, IL-8, and IL-10 were measured using an immunofluorescence kit designed for cytokine combination assays (BD FACS Canto II, the United States). D-dimer levels were evaluated prior to surgery and one hour after discontinuation of CPB.

Secondary outcomes included postoperative composite morbidity-mortality metrics; analysis of nine additional inflammatory cytokines (IL-1, IL-2, IL-4, IL-5, IL-12, IL-17, TNF-α, IFN-α, and IFN-γ) conducted by the hospital’s laboratory; the duration of mechanical ventilation (MV); and the length of stay (LOS) in both the PICU and the hospital. These composite outcomes encompassed in-hospital mortality, respiratory infection, cardiac arrest, the use of extracorporeal membrane oxygenation (ECMO), acute kidney injury (AKI), low cardiac output syndrome (LCOS), and prolonged MV (over 72 hours). LCOS was identified by clinical signs such as tachycardia, oliguria, and cold extremities, necessitating additional pharmacological or other supportive measures within the first 36 hours [22]. AKI was defined as the pediatric Risk, Injury, Failure, Loss, End Stage Renal Disease (pRIFLE) criteria being at least at the “Injury” stage [23]. The VIS was recorded upon PICU admission at intervals of 6, 12, 18, 24, and 48 hours post-surgery. Additionally, postoperative blood glucose and lactate levels, along with insulin treatment, were documented.

### 2.5 Statistical Analysis

Based on a prior study [9], an estimated 82 patients were calculated to be sufficient to identify a significant difference in IL-6 levels between the two groups. To account for potential dropouts, the sample size was increased by 5%, resulting in a total of 88 patients (44 in each group). This sample size of 88 is also consistent with that used in similar previous studies examining the effects of MP in neonatal cardiac surgery [18].

Categorical data are reported as frequencies and percentages. We treated the composite outcome as a binary variable, defined as the occurrence of at least one event during the follow-up period. Continuous data are presented either as mean ± standard deviation or as median with interquartile range (IQR) spanning the 25th to 75th percentiles. The normality of the distributions was visually inspected using Q–Q plots and histograms. Depending on the data type and distribution, categorical variables were analyzed using either the chi-square test or Fisher’s exact test, while continuous variables were analyzed with a *t*-test or Mann–Whitney U test. Preplanned subgroup analyses for composite outcomes considered sex (male vs. female), body weight (≤2.5 kg vs. >2.5 kg), age (≤7 days vs. >7 days), duration of CPB (≤120 minutes, >120 minutes/≤180 minutes, vs. >180 minutes), and the use of deep hypothermic circulatory arrest (DHCA) (yes vs. no). A sensitivity analysis using multivariable logistic regression was performed to adjust for core confounders (CPB duration, preoperative IL-6 levels, and body weight) to evaluate the independent association between MP administration and composite outcomes. Statistical analyses were conducted using SPSS 25.0 (SPSS Inc., Chicago, IL, USA), with *p*-value < 0.05 indicating statistical significance.

## 3. Results

### 3.1 Study Participants

**Fig. 1.**
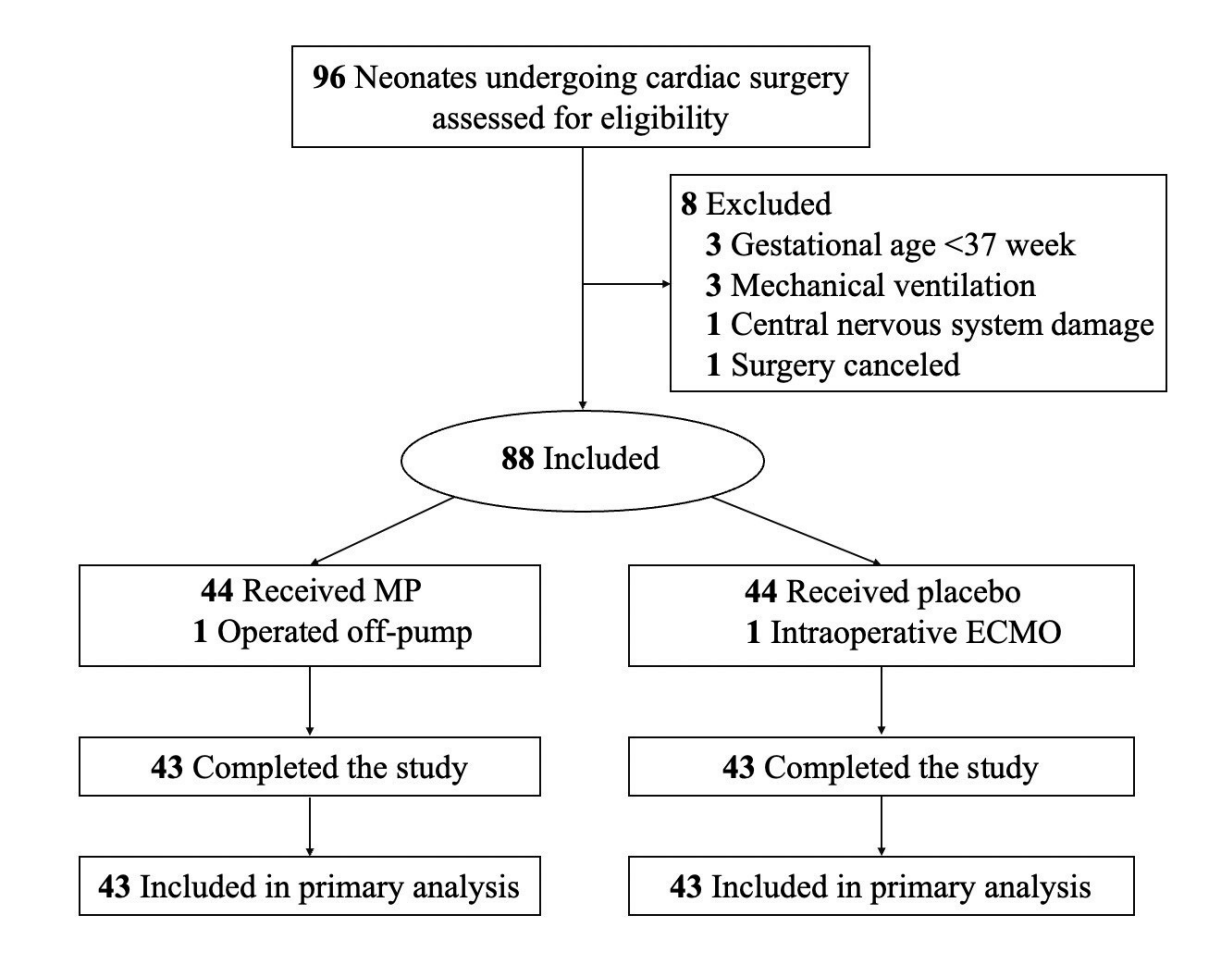
**Flow chart of the study**. MP, methylprednisolone; ECMO, extracorporeal membrane oxygenation.

Enrollment for the study was from December 2020 to August 2023. A total of 88 patients were included. Among these, 86 patients (43 in each group) received either MP or placebo (Fig. 1). Two patients were excluded from the analysis due to operated off-pump or receiving intraoperative ECMO. Patient demographics, cardiac diagnoses, and clinical characteristics are detailed in Table 1. Both the MP and placebo groups were comparable in terms of age, sex, weight, and type of surgical procedure performed. The average age at the time of surgery was 14.30 ± 8.12 days, with an average weight of 3.26 ± 0.56 kg, and a higher proportion of male patients (65.1% compared to 34.9%). The most frequent cardiac conditions included transposition of the great arteries, aortic arch hypoplasia, and total anomalous pulmonary venous connection, which represented 90% of the cases. Furthermore, variables such as gestational age, birth weight, height, body mass index, duration of CPB, duration of aortic cross-clamping, usage and duration of DHCA, and the lowest nasopharyngeal/rectal temperatures during CPB were similar between the groups.

**Table 1.** **Patient demographic and clinical characteristics**.

		Methylprednisolone (n = 43)	Placebo (n = 43)	*p *value
Age at surgery (days)		13.67 ± 7.42	14.93 ± 8.80	0.953
Sex (male)		29 (67.4%)	27 (62.8%)	0.651
Gestational age (weeks)		38.41 ± 1.05	38.53 ± 1.38	0.686
Birth weight (kg)		3.11 ± 0.48	3.08 ± 0.60	0.819
Weight (kg)		3.23 ± 0.48	3.29 ± 0.63	0.618
Height (cm)		49.81 ± 3.39	49.86 ± 3.96	0.953
Body mass index (kg/m^2^)		13.02 ± 1.53	13.21 ± 2.03	0.619
Diagnosis				0.723
	Transposition of the great arteries	13 (30.2%)	16 (37.2%)	
	Aortic arch hypoplasia	12 (27.9%)	11 (25.6%)	
	Total anomalous pulmonary venous connection	13 (30.2%)	12 (27.9%)	
	Truncus arteriosus	4 (9.3%)	3 (7.0%)	
	Pulmonary atresia	1 (2.3%)	1 (2.3%)	
CPB duration (min)		137.53 ± 47.07	138.21 ± 35.05	0.940
Aortic cross-clamp duration (min)		82.72 ± 31.12	83.93 ± 27.51	0.849
Use of deep hypothermic circulatory arrest		14 (32.6%)	10 (23.3%)	0.336
Deep hypothermic circulatory arrest duration (min)		33.30 ± 8.46	31.5 ± 4.23	0.638
Nadir nasopharyngeal temperature during CPB (℃)		28.31 ± 2.40	28.07 ± 2.25	0.625
Nadir rectal temperature during CPB (℃)		29.42 ± 1.93	29.14 ± 1.80	0.476

CPB, cardiopulmonary bypass.

### 3.2 Laboratory Test

**Fig. 2.**
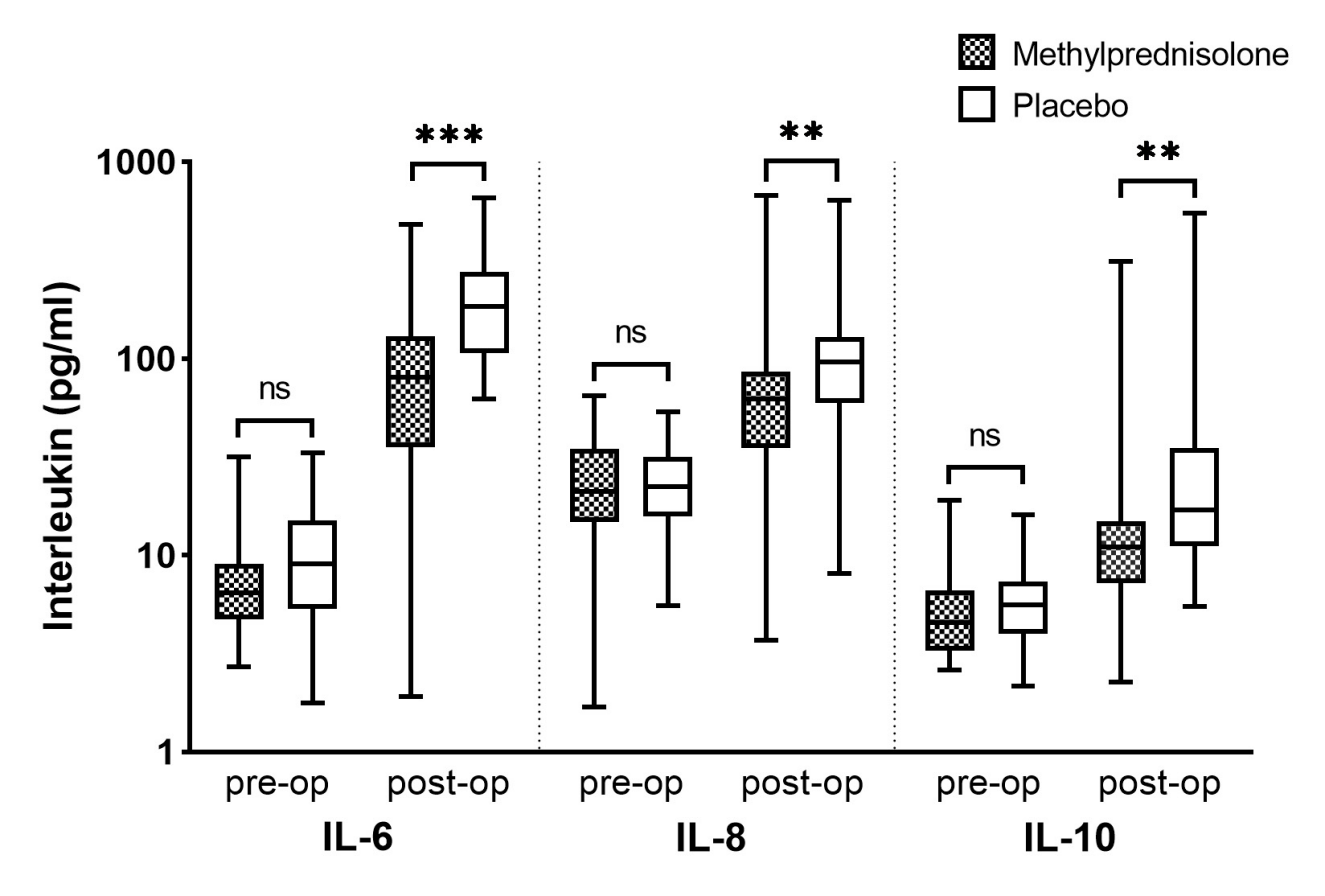
**Preoperative and postoperative plasma concentrations (median, interquartile range) of interleukin-6 (IL-6), IL-8, and IL-10 in the methylprednisolone group and placebo group**. NS, not significant (*p* > 0.05); ** represents *p* < 0.01; *** represents *p* < 0.001.

Fig. 2 illustrates that prior to surgery, the plasma concentrations of interleukins IL-6, IL-8, and IL-10 in the MP group were similar to those in the placebo group. The levels of these markers rose significantly the day after surgery, consistent with the anti-inflammatory effects of MP. Specifically, postoperative levels of IL-6 (median: 80.82, IQR: 43.32–145.64 vs. median: 175.37, IQR: 113.63–332.82, *p* < 0.001) and IL-8 (median: 67.77, IQR: 40.42–115.39 vs. median: 113.18, IQR: 75.89–133.68, *p* = 0.002) were significantly lower in the MP group compared to the placebo group. Additionally, the postoperative concentration of IL-10 was notably higher in the MP group (median: 14.77, IQR: 9.95–25.31 vs. median: 11.03, IQR: 8.84–14.54, *p* = 0.001). Although there was an increase in D-dimer levels following cardiac surgery, there were no significant changes in the pre- and postoperative plasma D-dimer concentrations between the two groups.

Baseline and postoperative laboratory data are detailed in Table 2. Initially, there were no significant differences between the two groups in terms of liver/kidney function or infection markers such as total bilirubin, aspartate aminotransferase (AST), alanine aminotransferase (ALT), creatine kinase isoenzyme (CK-MB), or high-sensitivity C-reactive protein (hsCRP). However, postoperative analysis showed that the MP group exhibited marginally higher levels of high-sensitivity C-reactive protein (median: 12.19, IQR: 10.77–12.97 vs. median: 11.71, IQR: 11.29–12.28, *p* = 0.080). Notably, the postoperative procalcitonin level was significantly higher in the MP group (median: 3.09, IQR: 0.95–8.36 vs. median: 0.27, IQR: 0.14–2.52, *p* = 0.009), although there was no significant difference in the serum N-terminal pro-brain natriuretic peptide levels between the groups.

**Table 2.** **Perioperative laboratory test data of the patients**.

	Methylprednisolone (n = 43)	Placebo (n = 43)	*p *value
Preoperative biochemical markers			
Total bilirubin (μmol/L)	100.20 (65.88–162.81)	121.68 (82.59–158.64)	0.962
AST (IU/L)	34.0 (27.0–42.0)	32.0 (25.50–46.50)	0.938
ALT (IU/L)	10.0 (7.0–14.0)	9.0 (5.50–17.0)	0.519
Serum creatinine (μmol/L)	31.70 (23.98–53.33)	36.46 (25.14–46.47)	0.666
CK-MB (IU/L)	4.01 (2.32–5.62)	4.26 (2.89–5.08)	0.854
hsCRP (mg/L)	0.48 (0.17–1.26)	0.77 (0.20–1.45)	0.671
Preoperative inflammation and coagulation markers			
D-dimer (μg/mL)	0.89 (0.59–1.51)	0.80 (0.52–1.35)	0.482
IL-6 (pg/mL)	6.45 (4.74–9.08)	9.36 (5.46–14.97)	0.104
IL-8 (pg/mL)	21.0 (14.79–30.63)	22.24 (15.91–31.54)	0.650
IL-10 (pg/mL)	4.56 (3.26–6.60)	5.63 (4.10–7.31)	0.112
IL-1 (pg/mL)	1.61 (1.15–2.25)	2.36 (1.47–3.45)	0.034
IL-2 (pg/mL)	1.51 (1.12–1.77)	1.91 (1.34–2.79)	0.002
IL-4 (pg/mL)	1.81 (1.09–2.59)	1.97 (1.14–2.54)	0.650
IL-5 (pg/mL)	1.36 (1.11–1.72)	1.59 (1.21–2.37)	0.077
IL-12 (pg/mL)	1.79 (1.16–2.63)	2.33 (1.52–3.37)	0.027
IL-17 (pg/mL)	1.81 (0.17–4.81)	4.16 (0.94–11.18)	0.016
TNF-α (pg/mL)	1.56 (1.06–2.17)	1.89 (1.26–4.12)	0.124
IFN-α (pg/mL)	1.59 (1.06–2.17)	2.50 (1.48–4.46)	0.003
IFN-γ (pg/mL)	1.65 (1.20–2.21)	1.98 (1.02–3.17)	0.361
Postoperative biochemical markers			
Total bilirubin (μmol/L)	146.97 (83.49–205.39)	151.14 (111.97–213.43)	0.819
AST (IU/L)	81.0 (71.0–119.5)	91.0 (79.0–142.0)	0.471
ALT (IU/L)	14.0 (10.0–26.0)	15.0 (8.0–31.0)	0.802
Serum creatinine (μmol/L)	63.18 (51.34–77.17)	56.99 (45.49–77.86)	0.799
CK-MB (IU/L)	31.56 (19.07–44.07)	36.12 (25.99–48.01)	0.487
hsCRP (mg/L)	12.19 (10.77–12.97)	11.71 (11.29–12.28)	0.080
NT-proBNP (pg/mL)	12431 (8756–30760)	10701 (8188–18912)	0.544
Procalcitonin (ng/mL)	3.09 (0.95–8.36)	0.27 (0.14–2.52)	0.009
Postoperative inflammation and coagulation markers			
D-dimer (μg/mL)	1.49 (0.94–2.14)	1.62 (1.26–2.27)	0.615
IL-6 (pg/mL)	80.82 (43.32–145.64)	175.37 (113.63–332.82)	<0.001
IL-8 (pg/mL)	67.77 (40.42–115.39)	113.18 (75.89–133.68)	0.002
IL-10 (pg/mL)	14.77 (9.95–25.31)	11.03 (8.84–14.54)	0.001
IL-1 (pg/mL)	1.89 (1.22–2.30)	1.70 (1.19–2.67)	0.870
IL-2 (pg/mL)	1.49 (1.23–1.77)	1.87 (1.52–2.48)	0.004
IL-4 (pg/mL)	1.76 (1.30–2.30)	1.45 (1.01–2.34)	0.235
IL-5 (pg/mL)	1.18 (1.00–1.83)	1.34 (0.99–2.30)	0.439
IL-12 (pg/mL)	2.14 (1.11–2.76)	1.79 (1.06–2.88)	0.580
IL-17 (pg/mL)	3.68 (1.35–5.32)	2.99 (0.81–6.90)	0.756
TNF-α (pg/mL)	1.85 (1.22–2.44)	2.04 (1.18–3.25)	0.490
IFN-α (pg/mL)	1.62 (1.22–2.68)	1.85 (1.30–2.91)	0.204
IFN-γ (pg/mL)	1.32 (1.12–2.03)	1.51 (1.18–2.12)	0.522

AST, aspartate aminotransferase; ALT, alanine aminotransferase; CK-MB, creatine kinase isoenzyme; hsCRP, high-sensitivity C-reactive protein; NT-proBNP, N-terminal pro-brain natriuretic peptide.

The differences in the levels of the other nine inflammatory cytokines during the perioperative period were not significant. Despite initially significant differences in IL-1, IL-2, IL-12, IL-17, and IFN-α levels before surgery, these disparities were narrowed or even eliminated postcardiotomy.

### 3.3 Clinical Outcomes

The clinical outcomes for the patients are detailed in Table 3. In summary, the composite outcome was observed in 25 patients (58.1%) in the MP group and 23 patients (53.5%) in the placebo group, with MP not significantly altering the odds did not detect a statistically significant improvement of the composite outcome (OR = 1.208; 95% CI: 0.515–2.832; *p* = 0.664), which was confirmed by sensitivity analysis (OR = 1.052, 95% CI: 0.403–2.761; *p* = 0.9172). There were no significant differences between the two groups in the individual components of the composite outcome. One patient in the placebo group passed away during hospitalization. AKI was reported in 16 patients (37.2%) in the MP group and 13 patients (30.2%) in the placebo group (*p* = 0.494). LCOS occurred in 8 patients (18.6%) in the MP group and 5 patients (11.6%) in the placebo group (*p* = 0.366). Prolonged mechanical ventilation (MV) over 72 hours was administered to 15 patients (34.9%) in the MP group and 17 patients (39.5%) in the placebo group, with no significant difference (*p* = 0.655). Respiratory infections were reported in 10 patients (23.3%) in the MP group and 9 patients (20.9%) in the placebo group (*p* = 0.795). There were no instances of cardiac arrest or use of extracorporeal membrane oxygenation (ECMO).

**Table 3.** **Postoperative clinical outcomes**.

	Methylprednisolone (n = 43)	Placebo (n = 43)	*p *value
Composite outcome	25 (58.1%)	23 (53.5%)	0.664
Components of the composite outcome			
Death	0	1 (2.3%)	0.237
Respiratory infection	10 (23.3%)	9 (20.9%)	0.795
Cardiac arrest/ECMO	0	0	-
Acute kidney injury	16 (37.2%)	13 (30.2%)	0.494
Low cardiac output syndrome	8 (18.6%)	5 (11.6%)	0.366
Prolonged MV (>72 h)	15 (34.9%)	17 (39.5%)	0.655
Other outcomes			
Peritoneal dialysis	11 (25.6%)	16 (37.2%)	0.245
Delayed sternal closure	5 (11.6%)	3 (7.0%)	0.456
Duration of MV (h)	49 (27–170)	50 (27.5–97.5)	0.644
Length of PICU stay (day)	18 (13–27.25)	18 (12.5–25.5)	0.736
Length of hospital stay (day)	24 (19–34)	25 (18–31)	0.986
RBC consumption (U)	1 (0.88–2)	1 (0.50–2.25)	0.940
Urine output on POD1 (mL)	389.53 ± 117.51	389.09 ± 128.68	0.986
Urine output on POD2 (mL)	584.19 ± 121.01	560.14 ± 161.65	0.457
Urine output on POD3 (mL)	526.56 ± 127.42	531.0 ± 170.44	0.891
Chest drainage on POD1 (mL)	45.16 ± 23.04	42.94 ± 21.20	0.643
Chest drainage on POD2 (mL)	15.16 ± 7.57	15.24 ± 8.32	0.962
Chest drainage on POD3 (mL)	10.93 ± 6.63	10.66 ± 7.20	0.858
VIS at PICU arrival	14.11 ± 4.02	17.26 ± 3.93	<0.001
Highest VIS at the first 24 h	17.56 ± 5.84	21.94 ± 5.66	0.001
Highest lactate at the first 24 h (mmol/L)	3.77 ± 1.87	3.44 ± 1.35	0.357
Highest blood glucose at the first 24 h (mmol/L)	12.23 ± 2.97	12.21 ± 2.78	0.982
Insulin administration	19 (44.2%)	14 (32.6%)	0.268
Nadir SvO_2_ at the first 24 h (%)	61.11 ± 10.67	55.94 ± 10.98	0.030

ECMO, extracorporeal membrane oxygenation; MV, mechanical ventilation; PICU, pediatric intensive care unit; RBC, red blood cell; POD, postoperative day; VIS, vasoactive inotropic score; SvO_2_, mixed venous oxygen saturation.

**Fig. 3.**
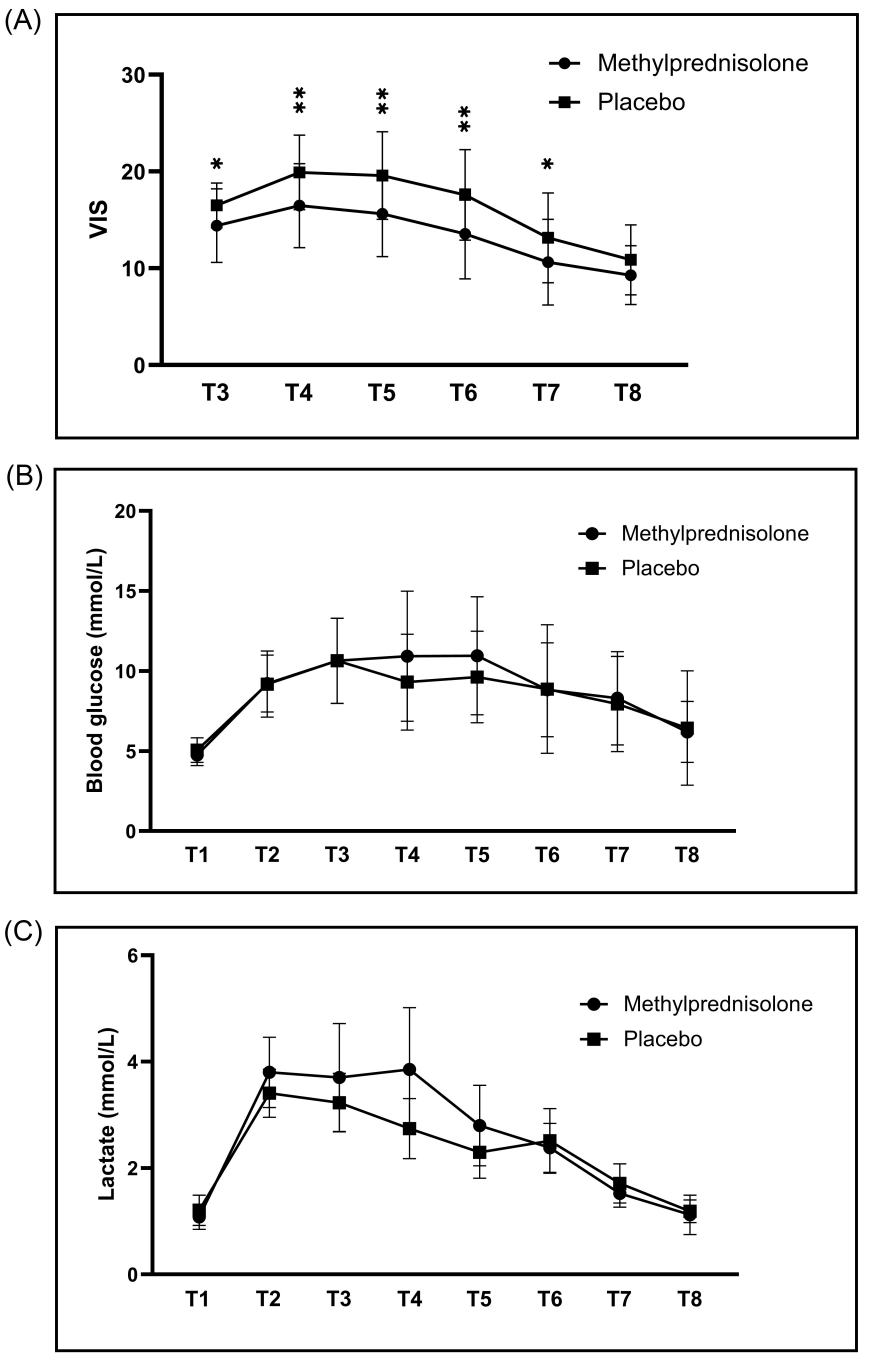
**Perioperative clinical markers between groups**. The VIS (A) and plasma concentrations (mean ± SD) of blood glucose (B) and lactate (C) levels in the methylprednisolone group and placebo group at different time points: T1, after anesthesia induction before administration of methylprednisolone; T2, highest value during surgery; T3, at PICU arrival; T4, 6 hours after PICU arrival; T5, 12 hours after PICU arrival; T6, 18 hours after PICU arrival; T7, 24 hours after PICU arrival; T8, 48 hours after PICU arrival. VIS, vasoactive inotropic score; PICU, pediatric intensive care unit. * represents *p* < 0.05, ** represents *p* < 0.01.

Secondary outcomes such as peritoneal dialysis, delayed sternal closure, duration of MV, length of stay in the PICU and hospital, urine output, chest drainage, and RBC consumption showed no significant differences between the MP and placebo groups. According to Fig. 3 and Table 3, MP was associated with a lower VIS at PICU arrival (14.11 ± 4.02 vs. 17.26 ± 3.93, *p* < 0.001) and the highest VIS during the first 24 hours postoperative was also lower in the MP group (17.56 ± 5.84 vs. 21.94 ± 5.66, *p* = 0.001). Moreover, the nadir mixed venous oxygen saturation (SvO_2_) on postoperative day one was significantly higher in the MP group (61.11 ± 10.67 vs. 55.94 ± 10.98, *p* = 0.030). No significant difference was observed in postoperative blood glucose levels between the two groups; the highest glucose level within the first 24 hours post-surgery was 12.23 ± 2.97 mmol/L in the MP group and 12.21 ± 2.78 mmol/L in the placebo group (*p* = 0.982). The incidence of insulin administration for hyperglycemia was higher in the MP group, though not significantly so (44.2% vs. 32.6%, *p* = 0.268). Likewise, the highest lactate levels during the initial 24-hour postoperative period showed no significant difference between the groups (3.77 ± 1.87 vs. 3.44 ± 1.35, *p* = 0.357).

### 3.4 Subgroup Analysis

MP showed no differential effects on the composite outcome among the subgroups, as indicated in Table 4. Subgroup interaction analysis showed no significant interaction between body weight subgroups and MP intervention (*p* = 0.3803), indicating that the effect of MP on composite outcomes was consistent across different body weight subgroups.

**Table 4.** **Subgroup analyses of the composite outcome in the methylprednisolone and placebo groups**.

		Methylprednisolone		Placebo		Odds Ratio (95% CI)	*p* Value
		No. Events	No. Subjects	No. Events	No. Subjects		
Overall		25	43	23	43	1.208 (0.515, 2.832)	0.664
Sex							
	Male	19	29	13	27	2.046 (0.698, 5.997)	0.192
	Female	6	14	10	16	0.450 (0.104, 1.946)	0.285
Weight							
	≤2.5 kg	3	4	1	3	6.000 (0.221, 162.531)	0.287
	>2.5 kg	22	39	22	40	1.059 (0.436, 2.573)	0.900
Age							
	≤7 days	6	13	6	9	0.429 (0.073, 2.500)	0.346
	>7 days	19	30	17	34	1.727 (0.634, 4.703)	0.285
Duration of cardiopulmonary bypass							
	≤120 min	6	12	9	18	1.000 (0.232, 4.310)	1.000
	>120 min/≤180 min	16	24	13	21	1.231 (0.362, 4.182)	0.739
	>180 min	3	7	1	4	2.250 (0.149, 33.933)	0.558
Deep hypothermic circulatory arrest							
	Yes	11	14	7	10	1.571 (0.245, 10.093)	0.634
	No	14	29	16	33	0.992 (0.365, 2.691)	0.987

## 4. Discussion

Despite ongoing technological advancements in CPB, the perioperative inflammatory response remains a significant challenge in neonatal cardiac surgery. This trial evaluated whether prophylactic MP (30 mg/kg) attenuates postoperative inflammation and improves clinical outcomes compared with placebo. MP administration following anesthesia significantly reduced pro-inflammatory cytokines (IL-6, IL-8) and increased anti-inflammatory IL-10 on postoperative day one, confirming its immunomodulatory effects. However, D-dimer levels—a marker of coagulation and fibrinolysis—did not differ between groups. Importantly, MP did not increase composite morbidity-mortality outcomes, including mortality, respiratory infections, cardiac arrest, ECMO requirement, AKI, LCOS, or prolonged mechanical ventilation (>72 hours) after adjusting for core confounders. Intraoperative MP was associated with a lower VIS, elevated procalcitonin levels, and improved nadir mixed venous oxygen saturation (SvO_2_) postoperatively.

Contemporary CPB innovations—such as biocompatible coatings, enhanced oxygenators, and ultrafiltration—alongside refined surgical techniques have reduced operative times and mitigated inflammation. Nonetheless, neonates, owing to their immature physiological systems and fragile endothelium, remain highly susceptible to CPB-induced inflammatory injury. Although our center utilizes optimized materials and dedicated surgical teams, inflammation persisted, and clinical outcomes remained comparable between groups. During CPB, contact between plasma proteases and non-endothelial surfaces activates the intrinsic coagulation pathway, triggering a cascade that generates pro-inflammatory byproducts. Ischemia-reperfusion injury and endotoxemia further amplify this response. Disseminated inflammation elevates serum IL-6 and IL-8, while leukocytes secrete anti-inflammatory cytokines such as IL-10. Inflammatory mediators interact with the vascular endothelium, inducing capillary leak syndrome—characterized by venous pooling, reduced systemic vascular resistance, impaired venous return, and hypotension. Moreover, these mediators directly depress myocardial function, predisposing neonates to postoperative ventricular dysfunction.

Achieving equilibrium between pro- and anti-inflammatory forces represents the primary goal of immunomodulation during CPB. Corticosteroids broadly suppress pro-inflammatory pathways while enhancing compensatory anti-inflammatory responses via glucocorticoid receptor binding and gene modulation. Keski-Nisula and colleagues [9,10,24] demonstrated superior anti-inflammatory effects with MP administered at anesthesia induction—findings consistent with our cytokine data. Prior studies suggested that corticosteroids attenuate coagulation activation, thrombin generation, and fibrinolysis [25,26]; however, we observed no intergroup differences in D-dimer levels, postoperative blood loss, or red blood cell transfusion, failing to corroborate a protective coagulation effect.

Although corticosteroid use is prevalent in pediatric cardiac surgery [27], practice patterns vary considerably due to insufficient evidence [15]. A multicenter survey reported that 96% of institutions utilize corticosteroids during pediatric CPB, yet only 40% administer them universally [27]. Suominen et al. [28] confirmed the safety of stress-dose corticosteroids in neonates without suppressing the hypothalamic-pituitary-adrenal axis. Nevertheless, recent large-scale randomized controlled trials have failed to demonstrate improved clinical outcomes with corticosteroids [16,29], and van Saet et al. [15] suggested that patient-specific factors may negate potential MP benefits. A meta-analysis of twelve randomized trials found no significant mortality reduction with corticosteroids; however, age-stratified analysis revealed that glucocorticoids may shorten mechanical ventilation duration in neonates [12,13]. Our study aligns with these mixed findings: MP did not influence composite morbidity-mortality or ventilation duration across any neonatal subgroup. Graham et al. [21] similarly reported no effect of 30 mg/kg MP on a composite endpoint (death, mechanical circulatory support, cardiac arrest, hepatic injury, AKI, hyperlactatemia) in 174 neonates. Likewise, the recent large-scale STRESS trial [17] showed that prophylactic MP failed to improve postoperative outcomes in infant cardiac surgery and was associated with increased hyperglycemia.

Hyperglycemia and heightened insulin requirements are well-recognized side effects of MP [9,28]. Although both groups exhibited postoperative glucose elevation, insulin use was marginally higher in the MP group—suggesting that MP may be clinically tolerable, though not definitively safe. Notably, postoperative VIS was significantly lower in the MP group, indicating a potential hemodynamic benefit. A meta-analysis of 17 pediatric studies (848 patients) concluded that corticosteroids improve VIS without affecting other clinical outcomes [30], and peak VIS serves as an early prognostic indicator in neonatal cardiac surgery, reflecting glucocorticoid-mediated vascular regulation [31].

CPB-induced inflammation contributes to postoperative pulmonary injury in congenital heart disease [32]. Our observation that MP improved nadir SvO_2_ on postoperative day one—without affecting lactate levels—may reflect reduced pulmonary exudation and enhanced oxygenation, despite unchanged systemic microcirculation. Although infection rates did not differ, elevated procalcitonin [33] in the MP group warrants vigilance for heightened infection risk.

The absence of demonstrable clinical benefit from MP may stem from the complexity and redundancy of the inflammatory cascade [34,35]. CPB elicits three distinct inflammatory phenotypes, each linked to disparate clinical trajectories [36]. Genetic polymorphisms likely modulate corticosteroid responsiveness in children undergoing CPB [37], and weight-based MP dosing yields substantial plasma level variability [38]. Future investigations should prioritize refined patient stratification to identify those most likely to benefit.

This study represents the largest single-center investigation to date evaluating prophylactic corticosteroid administration in neonatal cardiac surgery involving CPB.

### Limitations

The lack of a randomized controlled design and blinding protocol may introduce bias. This study was primarily designed to observe differences in plasma interleukin levels, which may mean it lacks sufficient statistical power to effectively evaluate the clinical benefits of MP and the relatively small sample size carries a potential risk of Type II error for secondary outcomes. This limitation is common in similar studies. The small number of neonatal cardiac surgeries makes it challenging to achieve an adequate sample size. Furthermore, the conclusions drawn may be less compelling because we did not measure the plasma concentrations of MP in this study.

## 5. Conclusions

In conclusion, preoperative administration of 30 mg/kg of MP may reduce the inflammatory response in neonates undergoing cardiac surgery with CPB. However, the prophylactic use of MP did not detect a statistically significant improvement in composite morbidity-mortality outcomes after adjusting for core confounders, except for the postoperative VIS and the lowest observed oxygen saturation (nadir SvO_2_). Given the non-randomized study design and potential bias, these findings should be interpreted with caution.

## Data Availability

All data reported in this paper will be shared by the lead contact upon request.

## Abbreviations

CPB, cardiopulmonary bypass; MP, methylprednisolone; AST, aspartate aminotransferase; ALT, alanine aminotransferase; CK-MB, creatine kinase isoenzyme; hsCRP, high-sensitivity C-reactive protein; NT-proBNP, N-terminal pro-brain natriuretic peptide; ECMO, extracorporeal membrane oxygenation; MV, mechanical ventilation; PICU, pediatric intensive care unit; RBC, red blood cell; POD, postoperative day; VIS, vasoactive inotropic score; SvO_2_, mixed venous oxygen saturation.
